# Economic cost of management of glaucoma in public and private health facilities in the Tema metropolis in Ghana

**DOI:** 10.4314/gmj.v58i1.4

**Published:** 2024-03

**Authors:** Matilda Adda, Samuel Amon, Justice Nonvignon, Moses Aikins, Genevieve C Aryeetey

**Affiliations:** 1 Ghana Health Service, Achimota Hospital, P.O. Box AH 15, Achimota, Accra, Ghana; 2 Department of Health Policy, Planning, and Management, School of Public Health, College of Health Sciences, University of Ghana, Legon, Accra, Ghana

**Keywords:** Economic cost, direct cost, indirect cost, glaucoma, Ghana

## Abstract

**Objectives:**

This study sought to determine the economic cost of the management of glaucoma among patients seeking care in health facilities in Ghana.

**Design:**

A cross-sectional cost-of-illness (COI) study from the perspective of the patients was employed.

**Setting:**

The study was conducted in public and private eye care facilities in the Tema Metropolis of Ghana.

**Participants:**

About 180 randomly selected glaucoma patients seeking healthcare at two facilities participated in the study.

**Main outcome measure:**

Direct cost, including medical and non-medical costs, indirect cost, and intangible burden of management of glaucoma.

**Results:**

the cost per patient treated for glaucoma in both facilities was US$60.78 (95% CI: 18.66-107.80), with the cost in the public facilities being slightly higher (US$62.50) than the private facility (US$ 59.3). The largest cost burden in both facilities was from direct cost, which constituted about 94% of the overall cost. Medicines (42%) and laboratory and diagnostics (26%) were the major drivers of the direct cost. The overall cost within the study population was US$10,252.06. Patients paid out of pocket for the frequently used drug- Timolol, although expected to be covered under the National Health Insurance Scheme (NHIS). Patients, however, expressed moderate intangible burdens due to glaucoma.

**Conclusion:**

The cost of the management of glaucoma is high from the perspective of patients. The direct costs were high, with the main cost drivers being medicines, laboratory and diagnostics. It is recommended that the National Health Insurance Authority (NHIA) should consider payment for commonly used medications to minimize the burden on patients.

**Funding:**

None declared.

## Introduction

Glaucoma is one of the leading causes of blindness globally (second to cataract),^1^ affecting about 67 million people^2^ and the number may reach 111.8 million in 2040.^1^ In 2020, it is estimated that about 11.2 million persons globally will be blind due to glaucoma.^3^ The disease accounts for about 15% of blindness in Africa, which is the region with the highest prevalence of blindness resulting from glaucoma.^2^-^4^ In West Africa, the most prevalent type of glaucoma is Primary Open Angle Glaucoma (POAG). Ghana has the highest prevalence of glaucoma in Africa and ranks second in the world.^5^ In 2015, it was reported that an estimated 700,000 Ghanaians were affected with glaucoma, with a sizeable number going blind for lack of treatment.

Regrettably, about 40% of those affected with glaucoma are not aware they have the disease. The inadequacy of eye screening exercises, together with poor information, contributed greatly to poor knowledge and inadequate awareness among individuals.^6^

In 1995, the Government of Ghana (GOG) launched the vision 2020 with the “Right to Sight” as one of its components. This initiative aimed at the elimination of blindness but stopped short of considering glaucoma.^7^ A major challenge in the management of glaucoma in Ghana is the cost of treatment. Stakeholders (patients, physicians, pharmacists, etc.) have complained about the high cost of treatment, particularly drugs, which are reported to be expensive due to taxation.^8^

Even though the Government of Ghana (GOG) introduced the National Health Insurance Scheme (NHIS) in 2003 with the primary aim of expanding access to healthcare via the removal of financial barriers, glaucoma patients continue to bear a significant financial burden. This is because the list of medications used to treat glaucoma under the scheme is limited. The list excludes more effective and potent drugs such as latanoprost.^8^,^9^ Since the NHIS does not cover the most effective and often expensive glaucoma medications, patients do rely on cheap and ineffective drugs.^10^

Furthermore, the cost of tests such as Visual Field Tests (VFT) and Nerve Fiber Layer Assessment (NFLA) are not covered by NHIS.^11^ This implies that the glaucoma patients in Ghana are faced with an enormous financial burden, including those with NHIS coverage. Other studies have estimated the economic cost of glaucoma.^12^,^13^ Some studies have concluded that, glaucoma patients are very likely to suffer the intangible burden, including psychological pain of anxiety and depression.^14^,^15^ The heavy economic cost combined with the fear of blindness and permanent visual impairment exacerbate the psychological problems glaucoma patients go through.^15^ However, though Ghana ranks second worldwide in terms of the prevalence of glaucoma, there is a paucity of economic cost estimates in Ghana. Hence, this study aimed to estimate the economic costs of managing glaucoma in Tema General Hospital Eye Clinic and Christian Eye Center, both in the Tema Metropolis of Ghana.

## Methods

### Study Design and Settings

This quantitative study employed a cross-sectional cost-of-illness design to estimate the economic cost of glaucoma from the perspective of households. The study was carried out at the Tema General Hospital Eye Clinic (TGH) and Tema Christian Eye Centre (TCEC), in May 2017. Tema General Hospital Eye Clinic is the only public health facility in the Tema Metropolis that offers primary, secondary, and tertiary specialist services in eye care and the TCEC is one of the few private eye specialist hospitals in the Tema Metropolis. The geographical location of TGH makes it a major referral point for all other public and private health facilities in and around the Metropolis, and the eye clinic attends to an average of 940 patients a month of which about 140 (14.9%) are glaucoma patients. TCEC offers medical, surgical, and optical eye services and attends to an average of 946 patients a month of which 165 (17.4%) are glaucoma patients.

### Study population and sampling

The study population was made up of glaucoma patients who attended public (Tema General Hospital Eye Clinic) and private (Tema Christian Eye Center) facilities in Tema. The two facilities were purposively selected because, comparatively, they are well-equipped and provide comprehensive eye care services. Cochran's formula was used to calculate the sample size.^16^ The sample size obtained for the study was 180. Since the study was conducted in two different facilities, the total number of participants from each facility was determined based on the proportion to the size of glaucoma patients attending the two health facilities. The TGH attends to an average of 140 glaucoma patients a month, whilst TCEC attends to an average of 165 glaucoma patients a month. Thus, based on these proportions, 82 patients were sampled from TGH and 98 patients from TCEC. A simple random sampling method was used to select glaucoma patients from the two facilities. By this, the outpatient register for each day within the one-month period of data collection was used as sample frame. Based on the frame, every second patient registered and seeking services was sampled for interview until the required sample size was reached. When a selected patient is unavailable or refuse to participate, the next patient on the register was selected for the exit interview.

### Sample size determination

Given that the outcome variable of interest is continuous, Cochran's sample size determination of means formula was used to calculate the sample size.^16^ Based on published literature, an estimated standard deviation of 0.327,^17^ was used, and a 95% confidence level with a sampling error of 0.05 or 5%.^13^

The estimated sample size was 164. Adjusting for a 10% non-response rate, the final sample size used for the study was 180 glaucoma patients who were willing and gave consent to participate in the study.

### Data collection

Data were collected by a trained Research Assistant (medical student), supervised by the first author – who is a practicing specialist Optometrist. During data collection, the Principal Investigator supervised Research Assistants and thoroughly crosscheck data collected for completeness, accuracy and consistency at the end of each day of data collection. The data collection tool was pretested through cognitive interviews with patients at the Greater Accra Regional Hospital.

Data were collected through exit interviews over one month from the two facilities using validated closed-ended paper-based questionnaires, produced by the authors based on reviewed literature on the topic. The questionnaire consisted of four main parts, namely: socio-demographic characteristics of glaucoma patients; direct treatment costs; indirect costs, and intangible burden components. The questionnaire was in English and interviewer administered.

Data on direct medical cost (i.e. registration, consultation, laboratory and diagnosis, medicine and surgery) were obtained from the health facilities pharmacy based on information retrieved from patients' folders, whereas direct non-medical cost data were verbally reported. All costs data focused on the past one-month duration of care. Furthermore, a five dimension Likert scale was used to elicit a response for the intangible burden. By this, glaucoma patients were asked to rate statements in respect of fear, emotional suffering, social isolation, and depression using the scales: (1) ‘Not at all’ (2) ‘A little’ (3) ‘Moderately’ (4) ‘Quite a bit’ (5) ‘Extremely’.

### Data analysis and cost estimation

Costs were analyzed from the household perspective and over one month preceding data collection. Three cost types were determined i.e., direct cost, indirect cost, and intangible burden. STATA version 15 and Microsoft Excel 2016 were used for data analyses. Table 1 shows details of the cost analysis. Statistical significance in cost difference was determined using Kruskal-Wallis and Wilcoxon Rank Sum tests. The significance level was set at 0.05.

**Table 1:** Cost analysis of glaucoma healthcare management

Cost type	Cost component	Cost Estimation Approach
**Direct**	Medical	*Consultation:* This was calculated by summing cost incurred on registration and consultation by patient. *Medicine:* This was calculated by summing the cost incurred by patients on drugs prescribed. *Diagnostics:* This was calculated by summing the cost incurred by patients on diagnostics test for patients. Surgery: This was calculated by summing cost incurred by patients on surgery that patient underwent. *Total medical cost:* This was estimated by summing total cost incurred by glaucoma patients on consultation, diagnostics, medication and surgery.
	Non-medical	*Travel:* This was calculated by summing all transportation cost incurred by patients and their caregivers for travelling to and from the eye clinic. *Food:* This was calculated by summing the costs incurred by patients and their caregivers on food items, including beverages and water. *Miscellaneous:* This was calculated by summing all costs incurred by patients on items such as telephone calls, phone credits and other items purchased because of the patient's eye condition. *Total non-medical cost:* This was estimated by summing the total cost of travel, food and miscellaneous expenses incurred by glaucoma patients during the study period.
	***Total direct cost***	This was the summation of the total medical and total non-medical costs
**Indirect**	Productivity losses due to seeking glaucoma treatment	The human capital approach used by Amon and Aikins 18 was employed to estimate the productivity losses by patients. This was sum of work hours spent seeking treatment for the glaucoma condition (travel, waiting and treatment times)
	Other productivity losses	This was calculated by summing total number of other productive work hours lost to patients due to glaucoma management activities, other than treatment seeking.
	***Total indirect valuation***	This was estimated by multiplying total productive hours lost (i.e., seeking health care and other productivity losses) by Ghana's average hourly earnings of US$2.02 per day (Ghana Ministry of Finance, June 2017).
**Total Cost**	***Total glaucoma management cost***	This was the summation of the total direct and total indirect costs
	***Sensitivity analysis***	The robustness of cost estimates was tested through one-way and multi-way sensitivity analyses. This was done by varying critical costs components of the data which lacked certainty (i.e., medications and wages) by 3%, 5% and 10%.
**Intangible burden**	Composite intangible scores	A composite intangible score was generated from responses to 5-dimension Likert scale questions adapted from validated tools, in relation to fear, emotional suffering, social isolation and depression due to the glaucoma illness. The aggregated score from the 5 dimensions were categorized into ‘low’ (16 – 37), ‘moderate’ (38 – 58) and ‘high’ (59 - 80) using the descriptive tertile statistics approach.^16^ Chi-square test and Fisher's exact tests were conducted to determine association between intangible burden and patients sociodemographic characteristics. Kruskal-Walis test and Wilcoxon Rank Sum test was used to test relationships between intangible scores for each domains and patient characteristics.

Descriptive analysis of categorical variables was examined using numbers and proportions whereas that of continuous variables was estimated using means and deviations. Equal variance and normality assumption of the numerical outcome of interest was tested using the Bartlett test and Shapiro-Wilk respectively. The socio-economic status of respondents was estimated based on asset ownership as proxy for income. Principal component analysis (PCA) was used to generate socioeconomic scores which were further classified into wealth quintiles.

### Ethical consideration

Ethical clearance for the study was obtained from the Ghana Health Service Ethical Review Committee of the research department of the Ghana Health Service (GHS-ERC: 130/02/2017). Permissions were obtained from the Tema Metropolitan Health Directorate, as well as TGH and TCEC administrations. Informed consent was acquired from sampled glaucoma patients and they were assured of confidentiality and privacy before they participated in the study.

## Results

Of the 180 glaucoma patients that participated in the study, 92 (51.1%) were males (see Table 2). The mean age of the respondents was 59 years. About 68% were married and 16.1% were uneducated. Overall 41.7% of patients in both facilities were employed and 46.1% were retired. Over 40% of study patients were within the poorer wealth quintile or below. Close to 91% of the patients were registered with the National Health Insurance Scheme (NHIS) of Ghana.

**Table 2:** Sociodemographic characteristics of participants

Characteristics	Private HF* N (%)	Public HF* N (%)	All facilities N (%)
**Sex**			
**Male**	50 (51.0)	42 (51.2)	92 (51.1)
**Female**	48 (49.0)	40 (48.8)	88 (48.9)
**Age:**			
**<40**	8 (8.1)	16 (19.5)	24 (13.3)
**40-59**	28 (28.6)	29 (35.4)	57 (31.7)
**60+**	62 (63.3)	37 (45.1)	99 (55.0)
**Marital status**			
**Married**	66 (67.3)	57 (69.5)	123 (68.3)
**Unmarried**	32 (32.7)	25 (30.5)	57 (31.7)
**Educational Level:**			
**No Education**	15 (15.3)	14 (17.1)	29 (16.1)
**Primary/Junior Secondary School**	23 (23.5)	27 (32.9)	50 (27.8)
**Secondary**	35 (35.7)	15 (18.3)	50 (27.8)
**Tertiary**	25 (25.5)	26 (31.7)	51 (28.3)
**Employment status:**			
**Employed**	35 (35.7)	40 (48.8)	75 (41.7)
**Unemployed**	6 (6.1)	8 (9.7)	14 (7.8)
**Student/apprentice**	3 (3.1)	5 (6.1)	8 (4.4)
**Retired**	54 (55.1)	29 (35.4)	83 (46.1)
**Wealth quintile**			
**Poorest**	12 (26.1)	9 (18.0)	21 (21.9)
**Poorer**	7 (15.2)	13 (26.0)	20 (20.8)
**Middle**	12 (26.1)	10 (20.0)	22 (22.9)
**Richer**	7 (15.2)	7 (14.0)	14 (14.6)
**Richest**	8 (17.4)	11 (22.0)	19 (19.8)
**NHIS status**			
**Enrolled**	88 (89.8)	75 (91.5)	163 (90.6)
**Not enrolled**	10 (10.2)	7 (8.5)	17 (9.4)

* HF: Health Facility

** US$1.00 is equivalent to GHS4.36 (Bank of Ghana average monthly interbank exchange rate for June, 2017)

### Direct and indirect costs

Table 3 shows that the cost per patient treated for glaucoma in both facilities was US$60.78 (95% CI: 18.66-107.80), with the cost in the public facilities being slightly higher (US$62.50) than the private facility (US$ 59.3).

**Table 3:** Total cost of glaucoma care

Cost component	Private facility (TCEC)		Public facility (TGH)		All facilities		
	Mean (95% CI)	Cost Profile	Mean (95% CI)	Cost Profile	Overall Total Cost	Mean (95% CI)	Cost Profile
	(US$)*	(%)	(US$)	(%)	(US$)	(US$)	(%)
**Direct Cost**							
***Medical Cost***							
**Registration/Consultation**	4.69 (4.22-5.16)	7.9	5.83 (5.06-6.60)	9.3	938.07	5.21 (4.64-8.46)	8.6
**Laboratory/diagnostics**	15.00 (7.07-22.91)	25.3	17.21 (5.58-28.83)	27.5	2451.15	16.11(6.33-25.87)	26.5
**Medicines**	24.70 (7.61-41.79)	41.7	27.06 (4.30-49.81)	43.3	4639.33	25.78 (5.96-45.80)	42.4
**Surgery**	229.36	8.3	-				
***Sub-total***	***44.39(18.90-69.86)***	***74.8***	***50.10 (14.94-85.24)***	***80.1***	***8487.27***	***47.10 (16.93-80.13)***	***77.4***
***Non-medical cost***							
**Travel**	7.47 (1.58-13.36)	12.6	5.73 (0.55-10.91)	9.2	1201.93	6.60 (1.06-12.14)	10.8
**Food**	2.35 (0-5.14)	3.9	1.71 (0.25-3.17)	2.7	100.69	2.03 (0.13-4.16)	3.3
**Drinks/water**	0.79 (0-1.61)	1.3	0.60 (0.11-1.08)	0.9	38.76	0.69 (0.06-1.35)	1.1
**Miscellaneous**	0.74 (0.08-1.40)	1.3	0.53 (0.17-0.88)	0.8	28.44	0.64 (0.10-1.13)	1.1
***Sub-total***	***11.35 (1.66-21.51)***	***19.1***	***8.57 (1.08-16.04)***	***13.7***	***1369.82***	***9.96 (1.35-18.78)***	***16.4***
**Total direct cost**	**55.74 (20.56-91.37)**	**93.9**	**58.67(16.02-101.28)**	**93.8**	**9857.09**	**57.06 (18.28-98.91)**	**93.9**
**Indirect Cost** **							
**Patients' valued lost time**	0.85 (0.13-1.58)	1.4	1.86 (0.63-3.09)	2.9	236.28	2.71 (0.38-2.34)	4.5
**Caregivers' valued lost time**	2.72 (0-6.03)	5.6	2.01 (0-7.06)	3.2	431.69	2.37 (0-6.55)	3.9
**Total Indirect Cost**	**3.57 (0.13-7.61)**	**6.0**	**3.87 (0.63-10.15)**	**6.2**	**667.98**	**3.72 (0.38-8.89)**	**6.1**
**TOTAL COST**	**59.31(20.6998.98)**	**100.0**	**62.54(16.65-111.43)**	**100.0**	**10,525.06**	**60.78(18.66-107.80)**	**100.0**

* USD1.00 is equivalent to GHS4.36 (Bank of Ghana average monthly interbank exchange rate for June, 2017)

** National minimum wage of USD2.02 per day was used to value loss productivity (Ministry of Finance, June, 2017)

The largest cost burden in the two facilities was from direct cost which constituted about 94% of the overall cost. Medicines (42%) and laboratory and diagnostics (26%) were the major drivers of the direct cost. The overall cost within the study population was US$10,252.06.

The estimated average indirect cost of glaucoma at the private and public hospitals were US$3.57 (95% CI:0.13-7.61) and US$3.87 (95% CI:0.63-10.15) respectively. Indirect cost contributed about 6% to the total cost burden of glaucoma, and the average indirect cost was US$3.72 (95% CI:0.38-8.89). Glaucoma patients in the public facility lost more than twice the productive hours lost by their counterparts in the private facility. Whilst the average waiting time was 4.79 hours in the public facility, it was only 1.12 hours in the private facility. Averagely, respondents enrolled on the NHIS incurred US$53.28 (95% CI:18.18-88.39), whereas those not enrolled on NHIS incurred US$68.92 (95% CI:0-149.46).. The direct and indirect cost profiles were approximately the same in both private and public facilities (i.e. 94% and 6% respectively).

One-way sensitivity analyses conducted by varying the cost of medication by 3%, 5%, and 7% yielded respectively 1.3%, 2.2%, and 3.1% increases in total cost. Conversely, the same analysis conducted on wage rate yielded percentage increases of 0.2, 0.3, and 0.4 respectively in total cost.

Meanwhile, whereas 3%, 5%, and 7% variations in medication respectively resulted in 0.1, 0.1, and 0.2 percentage gain in direct cost.

The same level of variations in wage rate respectively resulted in 0.2, 0.3, and 0.4 percentage increases in indirect cost. Furthermore, concurrent variations in both medication and wage rates by 3%, 5%, and 7% resulted in a percentage fall in direct cost proportion to the total treatment cost and thus a percentage rise in indirect cost in proportions to total treatment cost. However, overall, there were 1.5%, 2.6%, and 3.5% increases in total treatment cost respectively. The results of the sensitivity analysis showed that the cost estimates of this study were sensitive to changes in wage and medicine cost variables.

Further analysis of the relationship between patients' socioeconomic status and direct cost of glaucoma care show that private facility patients in the poorest wealth quintile with an average income of US$ 48.58 per month spent over 80% of their total monthly income on seeking glaucoma care (see Table 4).

**Table 4:** Direct cost burden by wealth quintile and facility type

Quintile	Private facility (TCEC)				Public facility (TGH)		
	Average income (US$)*	Average direct cost (US$)*	Proportion of cost burden to income		Average income (US$)*	Average direct cost (US$)*	Proportion of cost burden to income
**Poorest**	48.85	39.51	80.88		57.68	54.23	94.02
**Poorer**	104.49	50.36	48.20		111.81	54.87	49.07
**Middle**	157.68	54.49	34.56		165.14	65.60	39.72
**Richer**	221.71	56.81	25.62		262.61	71.28	27.14
**Richest**	545.36	54.03	9.91		550.46	68.92	12.52

* US$1.00 is equivalent to GHS4.36 (Bank of Ghana average monthly interbank exchange rate for June, 2017)

Comparatively, public facility patients spent over 94% of their total monthly income on glaucoma care. Patients of both facility levels in the richest quintile spent the lowest percentage of their income on seeking glaucoma care. Thus, patients of both facilities in the poorest wealth quintile suffered the greatest direct cost burden whilst those in the richest wealth quintile suffered the least cost burden.

Table 5 shows the relationship between the cost of glaucoma care and the background characteristics of patients. The cost of glaucoma care increases with the duration of care. There was a statistically significant relationship between the mean direct cost and educational level (p<0.05), and wealth status (p<0.05). Likewise, the total cost of glaucoma care had a statistically significant relationship with patient wealth status (p<0.05).

**Table 5:** Relationship between cost and background characteristics

Characteristic	Direct Cost (US$)		Indirect Cost (US$)		Total Cost (US$)	
	Mean (SD)	p-value	Mean (SD)	p-value	Mean (SD)	p-value
**Sex**						
**Male**	55.02 (38.29)	0.67	3.37 (4.64)	0.08	58.39 (39.05)	0.75
**Female**	54.49 (44.68)		4.07 (4.38)		58.56 (45.91)	
**Age**						
**< 40**	48.61 (24.61)	0.92	1.56 (1.25)	<0.001***	50.17 (24.75)	0.58
**40-59**	52.00 (28.16)		2.12 (2.35)		54.11 (28.46)	
**60+**	57.84 (50.16)		5.15 (5.38)		63.00 (5.38)	
**Marital Status**						
**Married**	52.31 (35.43)	0.37	2.90 (3.74)	0.001***	55.21 (36.33)	0.15
**Unmarried**	60.05 (52.06)		5.46 (5.49)		65.50 (52.95)	
**Educational Level**						
**No education**	54.91 (34.36)	0.02*	4.10 (3.35)	0.27	59.03 (34.54)	0.03*
**Primary/JSS**	44.43 (24.03)		4.70 (6.62)		49.13 (26.39)	
**Secondary**	52.94 (51.46)		3.26 (3.43)		56.21 (52.48)	
**Tertiary**	66.59 (45.48)		2.96 (3.21)		69.56 (46.64)	
**Employment Status**						
**Employed**	53.51 (27.17)	0.12	2.26 (3.67)	<0.001***	55.78 (28.06)	0.07
**Unemployed**	35.50 (16.67)		2.24 (2.45)		37.75 (17.34)	
**Student/apprentice**	45.87 (23.41)		2.17 (2.96)		48.05 (22.86)	
**Retired**	56.00 (53.75)		5.42 (5.02)		65.41 (54.65)	
**Wealth quintile**						
**Poorest**	43.15(26.83)	0.04*	2.87(3.54)	0.14	46.02(26.91)	0.06
**Poorer**	57.02(28.33)		3.72(3.12)		60.74(29.10)	
**Middle**	59.54(29.36)		3.13(3.34)		62.67(31.16)	
**Richer**	71.89(37.80)		2.05(2.80)		73.93(39.29)	
**Richest**	57.10(23.33)		1.65(1.76)		58.75(23.79)	
**NHIS status**						
**Enrolled**	53.28 (35.11)	0.67	3.78 (4.64)	0.42	57.07 (36.09)	0.84
**Not enrolled**	68.92 (80.54)		3.01 (3.11)		71.93 (82.05)	
**Length of treatment (Years)**						
**<1**	47.84 (26.26)	0.36	2.47 (2.48)	0.18	50.30 (26.41)	0.23
**1-5**	57.32 (51.00)		4.06 (4.76)		61.38 (52.18)	
**>5**	57.66 (40.76)		4.34 (5.30)		62.01 (41.82)	

* p < 0.05

** p < 0.01

*** p < 0.001 significant levels

Wilcoxon Rank Sum test used to determine statistical significance in mean difference of two categories

Kruskal-Wallis test used to determine statistical significance in mean difference of more than two categories

Also, a statistically significant relationship exists between indirect cost and age, employment status, marital status, and wealth status.

### Intangible burden

**Figure 1:**
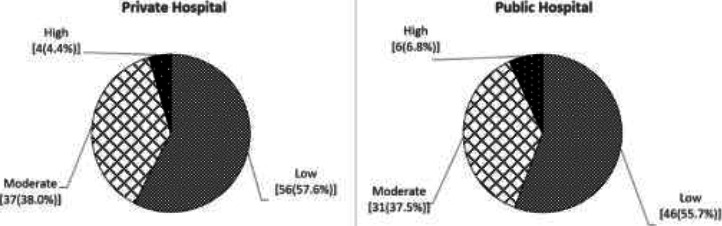
Intangible burden of glaucoma

Figure 1 shows that the intangible burden is similar in patients from both facilities. The high proportion of patients in both facilities reported a relatively low intangible burden. Further analysis of the relationship between intangible burden and background characteristics shows a statistically significant difference in mean score between social isolation and all of the background characteristics except sex, namely age (p<0.01), marital status (p<0.01), educational level (p<0.05), wealth status (p<0.01) and length of glaucoma care (p<0.01). Likewise, there was a statistically significant difference in the mean score between emotional pain and educational level (p<0.05), employment status (p<0.05), and length of glaucoma treatment (p<0.01). Furthermore, a statistically significant mean difference exists between depression and educational level (p<0.01), wealth status (p<0.01), and length of glaucoma treatment (p<0.05).

## Discussion

This study sought to determine the economic cost of glaucoma among patients seeking care in private and public health facilities. The total estimated cost of glaucoma care for a month was US$10,525.07, which translates into US$ 126,300.84 per annum. The direct cost constituted 93.7% of the cost profile. The average monthly cost of seeking glaucoma care was estimated to be US$58.47 (95% CI:16.05-58.47). Both public and private facilities recorded approximately the same total direct cost profile (94%). This was probably because the drugs (which is the main cost driver) prescribed in both facilities were similar, hence the cost profile of medication in both facilities did not vary significantly. Also, a statistically significant relationship exists between average direct cost and patient's wealth status.

The estimated average cost of seeking glaucoma care is much lower than a similar study conducted by Adio et al.,^2^ which reported a monthly average cost of close to double that estimated in this study. The estimated medical cost, which constitutes the highest cost profile in this study is slightly higher than that reported by Ocansey et al.^5^ The difference may be due to the difference in sample size and context. Consistent with findings from Denmark which indicated that medication cost constitutes the highest proportion (57%) of the total cost profile of glaucoma care,^19^ the cost of medicines in this study constitutes the greatest portion of the total cost (44.1%).

The estimated average monthly cost of medicines was considerably lower than that reported from Nigeria.^2^ The cost difference may be attributed to the considerably higher cost of living in Nigeria as compared to Ghana. The direct medical cost was comparable in both the private and public facilities. This may be because the same treatment guidelines are used in both facilities. Also, the same kind of diagnostic test is required irrespective of whether one attends a public or private facility. Consequently, patients in both facilities incur similar direct medical cost of managing glaucoma, particularly since most of the glaucoma medication is not covered under NHIS.^10^

Frequently used medicines by study participants include Timolol, Betaxolol, Levobunolol, Bexalol, Brimonidine, Betoptic, Betaxolol, Lantanoprost, Brinzolamide, Travaprost, Acetazolamide, Bimotoprost, Lexobunolol, Proximol, Dorzolamide, Prsotan, Brimonidine+Brinzolamide, Acetazolamide, Dorzolamide+Timolol, Travaprost+Timolol. Timolol, which is the cheapest and most frequently used drug by study respondents was not provided to insured patients, even though it is included in the medicines list under the NHIS. Both private and public facility patients pay for commonly used drugs out of pocket, whether or not they were enrolled on the NHIS The NHIS however paid for the consultation and registration fees for patients who attended the public facility. Similar to findings by Adio et al.,^2^ the cost of travel made up the bulk of the direct non-medical cost. However, the estimated cost of travel in this study was lesser.

These cost differences in the two studies may be attributed to contextual differences.

About 65% of the total indirect cost was productive caregivers' time lost. This could be attributed to the fact that 55% of the patients were over 60 years old and retired, hence reported minimal productive lost days. Most of the patients in that age group came to the facility with caregivers. This corroborates report from a global study by Varma et al. which suggested that late disease leads to greater indirect costs e.g. family/home help and rehabilitation costs.^13^ The waiting times in the public facility was significantly higher than that in the private facility, hence respondents in the public facility lost more valued productive hours due to long waiting time. This confirms a study by Atinga et al., that found that there are generally long patient waiting times in public health facilities as compared to their private health facilities.^20^

This study also revealed that patients in the poorest wealth quintile spent a very substantial proportion of their income managing their glaucoma condition. Hence, patients of poor socio-economic status rely heavily on family support for treatment. It is worth mentioning that patients in the poorest wealth quintile of this study earn higher than the current minimum wage in Ghana. This implies that patients who earn minimum wage or lower may spend their entire income on glaucoma care. This is a disturbing situation since it will inevitably lead to high levels of non-compliance, and hence the potential dire consequence of glaucoma such as blindness. This finding is similar to a study by Adio et al., who reported that low-income earners may spend all their monthly earnings on treatment for glaucoma.^2^

Similar to report by Varma et al.,^13^ this study findings revealed that the financial burden of glaucoma increases as disease severity increases with time. Furthermore, as noted by Guedes et al.,^21^ since glaucoma is of genetic origin and cannot be prevented, early detection and diagnosis is the most cost-effective way of managing glaucoma and preventing blindness. Consistent with report by Ocansey et al.,^5^ this study found a statistically significant difference between the cost burden of glaucoma care and wealth status. Emotional pain was the major driver of intangible burden suffered by glaucoma patients followed by fear. Depression was the least scored domain. This contradicts findings by Agorastos et al., who found that glaucoma caused depression and emotional pain such as anxiety.^22^ Another study by Varma et al. also suggested that the psychological burden of glaucoma increases as vision decreases, along with a growing fear of blindness, social withdrawal from impaired vision, and depression.^13^ The relatively low depression reported in this study may be attributed to the general religious beliefs of most Ghanaians in supernatural healing and lack of patients' knowledge about their disease conditions.

### Limitation of the Study

Limitations of this study include: (1) time analysis focused on the only one-month duration of care; and (2) estimation of indirect cost did not include productivity losses due to disability and presenteeism. These limitations notwithstanding, the study results can be used to inform evidence-based policy on NHIS tariffs for glaucoma and, formulation and prioritization of health intervention to achieve policy efficiency.

## Conclusion

Glaucoma poses a significant burden on patients since patients have to be on treatment for the rest of their lives. This burden increases as the disease progresses. The direct cost of glaucoma care is higher in both private and public health facilities, and constitutes a significant portion of the total cost, with medicines being the main cost driver. Glaucoma patients in the poorest wealth quintile suffered a higher cost burden compared to their counterparts with better socioeconomic status. Patients also suffer a significant intangible burden of fear and emotional pain notwithstanding the facility they seek care. To minimize the economic burden of managing the glaucoma illness, frequently used medicines must be covered by the national health insurance.
